# Detection of *Rickettsia* and *Ehrlichia* spp. in Ticks Associated with Exotic Reptiles and Amphibians Imported into Japan

**DOI:** 10.1371/journal.pone.0133700

**Published:** 2015-07-24

**Authors:** Masako Andoh, Akiko Sakata, Ai Takano, Hiroki Kawabata, Hiromi Fujita, Yumi Une, Koichi Goka, Toshio Kishimoto, Shuji Ando

**Affiliations:** 1 Department of Virology-1, National Institute of Infectious Diseases, Tokyo, Japan; 2 Laboratory of Veterinary Public Health, Joint Faculty of Veterinary Medicine, Kagoshima University, Kagoshima, Kagoshima, Japan; 3 Department of Bacteriology-1, National Institute of Infectious Diseases, Tokyo, Japan; 4 United Graduate School of Veterinary Science, Yamaguchi University, Yamaguchi, Yamaguchi, Japan; 5 Mahara Institute of Medical Acarology, Tokushima, Japan; 6 Laboratory of Veterinary Pathology, School of Veterinary Medicine, Azabu University, Kanagawa, Japan; 7 National Institute of Environmental Studies, Ibaraki, Japan; 8 Okayama Prefectural Institute for Environmental Science and Public Health, Okayama, Japan; University of Minnesota, UNITED STATES

## Abstract

One of the major routes of transmission of rickettsial and ehrlichial diseases is via ticks that infest numerous host species, including humans. Besides mammals, reptiles and amphibians also carry ticks that may harbor *Rickettsia* and *Ehrlichia* strains that are pathogenic to humans. Furthermore, reptiles and amphibians are exempt from quarantine in Japan, thus facilitating the entry of parasites and pathogens to the country through import. Accordingly, in the current study, we examined the presence of *Rickettsia* and *Ehrlichia* spp. genes in ticks associated with reptiles and amphibians originating from outside Japan. Ninety-three ticks representing nine tick species (genera *Amblyomma* and *Hyalomma*) were isolated from at least 28 animals spanning 10 species and originating from 12 countries (Ghana, Jordan, Madagascar, Panama, Russia, Sri Lanka, Sudan, Suriname, Tanzania, Togo, Uzbekistan, and Zambia). None of the nine tick species are indigenous in Japan. The genes encoding the common rickettsial 17-kDa antigen, citrate synthase (*gltA*), and outer membrane protein A (*ompA*) were positively detected in 45.2% (42/93), 40.9% (38/93), and 23.7% (22/93) of the ticks, respectively, by polymerase chain reaction (PCR). The genes encoding ehrlichial heat shock protein (*groEL*) and major outer membrane protein (*omp-1*) were PCR-positive in 7.5% (7/93) and 2.2% (2/93) of the ticks, respectively. The *p44* gene, which encodes the *Anaplasma* outer membrane protein, was not detected. Phylogenetic analysis showed that several of the rickettsial and ehrlichial sequences isolated in this study were highly similar to human pathogen genes, including agents not previously detected in Japan. These data demonstrate the global transportation of pathogenic *Rickettsia* and *Ehrlichia* through reptile- and amphibian-associated ticks. These imported animals have potential to transfer pathogens into human life. These results highlight the need to control the international transportation of known and potential pathogens carried by ticks in reptiles, amphibians, and other animals, in order to improve national and international public health.

## Introduction

Members of the genera *Rickettsia* and *Ehrlichia* are obligate intracellular bacteria belonging to the order Rickettsiales, in the Rickettsiaceae and Anaplasmataceae families, respectively. The reported incidence of rickettsial disease is increasing worldwide because of increased public health concern and improvement in diagnostic technology [1]. Rickettsial and ehrlichial diseases are often transmitted by ticks. Ticks carrying pathogens can infest a variety of hosts, including domestic animals as well as humans [2]; therefore, tick-borne disease is an important concern in both human and veterinary medicine. As the pathogens and ticks both exhibit differential host affinity [2], identification of relationships among the pathogenic microbes, ticks, and animals is important in understanding the occurrence of rickettsial and ehrlichial disease.

Reportedly, *Rickettsia*-carrying reptile-associated ticks can be pathogenic to humans [3–6]. *Rickettsia honei*, which is the causal agent of Flinders Island spotted fever in Australia, is carried by *Bothriocroton hydrosauri* (formerly *Aponoma hydrosauri*) ticks, which feed on snakes and lizards [3]. *Ixodes ricinus* has a broad host range and can be found on certain lizards (*Lacerta viridis*); these ticks reportedly carry *Anaplasma*, *Borrelia*, and *Rickettsia* in central European populations [4]. In Poland, *Anaplasmacetae* and *Borrelia* have been detected in ticks collected from lizards (*Lacerta agilis*) [5]. *Anaplasma phagocytophilum*, which causes the potentially fatal tick-borne disease human granulocytic anaplasmosis, was isolated from multiple species of snakes and lizards in the United States [6]. Although there have been several global reports of reptile-associated ticks carrying members of the pathogenic *Rickettsiaceae* and *Anaplasmataceae* families, there has been no such information reported for Japan. Several ticks that infest reptiles also show affinity to amphibians, e.g., *Amblyomma rotundatum* [7]. Therefore, amphibians also have potential to carry or transfer pathogenic Rickettsiaceae and Anaplasmataceae.

Large numbers of exotic animals are currently imported into Japan, with at least 500,000 reptiles and 8,000 amphibians imported annually since 2004 (Trade Statistics of Japan [http://www.customs.go.jp/toukei/]). Japan has quarantine regulations for animals that are potential reservoirs of infectious diseases, under the Rabies Prevention Law, Domestic Animal Infectious Disease Control Law, and Human Infectious Diseases Control Law. However, reptiles and amphibians are not subject to quarantine regulations in Japan; therefore, the parasites, microbes, and other pathogens carried by these imported animals can invade into the country and have potential to cause emerging infections. In the present study, we report the incidence of ticks carrying *Rickettsia* and *Ehrlichia* associated with reptiles and amphibians imported from other countries into Japan. We detected genes of known pathogens that have not been reported in Japan as well as new genes from organisms without pathogenic information.

## Methods

### Tick collection and host animals

Ticks were collected from snakes, tortoises, lizards, and frogs from July 2004 to May 2009 from the following sources: on-site in Japan at licensed pet stores with import permits; animals purchased from licensed suppliers or pet stores in Japan; and on-site in the country of origin when awaiting export. Ticks sourced from outside of Japan were ethanol-fixed prior to submission. Any purchased animals were used in unrelated studies, and the rest remained at the source pet store or breeding facility. No specific permission was required under national regulations for performing noninvasive procedures such as tick collection in imported animals available for commercial purchase. This study did not use endangered or protected species.

The reptiles and amphibians originated from 12 countries (Republic of Ghana, Hashemite Kingdom of Jordan, Republic of Madagascar, Republic of Panama, the Russian Federation, Democratic Socialist Republic of Sri Lanka, Republic of the Sudan, Republic of Suriname, United Republic of Tanzania, Republic of Togo, Republic of Uzbekistan, and Republic of Zambia). The species and numbers of animals sampled are listed in Table 1. Ticks were collected humanely by experienced personnel, with consideration of animal welfare. The collected ticks were then identified morphologically to determine species, sex, and developmental stage. For ticks that we were unable to identify morphologically, the tick mitochondrial 16S rRNA gene (mt-*rrs*) and 12S rRNA gene (*12S rDNA*) were sequenced to identify the species [8, 9].

**Table 1:** Tick population, host species, and PCR detection rates of the rickettsial genes.

				PCR-positive rate (%) (No. of positive/No. of tested)					
Country of origin	Host species*	Tick species	Stage**	*Rickettsia* 17-kDa	*Rickettsia gltA*	*Rickettsia ompA*	*Ehrlichia omp-1*	*Ehrlichia groEL*	*Anaplasma p44*
**Africa**									
Ghana	*Python regius* (1)	*Amblyomma latum*	M	0.0 (0/3)	0.0 (0/3)	0.0 (0/3)	0.0 (0/3)	0.0 (0/3)	0.0 (0/3)
			F	0.0 (0/1)	0.0 (0/1)	0.0 (0/1)	0.0 (0/1)	0.0 (0/1)	0.0 (0/1)
		*Amblyomma transversale*	M	83.3 (5/6)	50.0 (3/6)	0.0 (0/6)	0.0 (0/6)	0.0 (0/6)	0.0 (0/6)
			F	90.9 (10/11)	90.9 (10/11)	0.0 (0/11)	0.0 (0/11)	0.0 (0/11)	0.0 (0/11)
Madagascar	*Phelsuma dubia* (1)	*Amblyomma latum*	M	100.0 (1/1)	0.0 (0/1)	100.0 (1/1)	0.0 (0/1)	0.0 (0/1)	0.0 (0/1)
Sudan	*Geochelone paradalis* (U)	*Amblyomma sparsum*	M	0.0 (0/2)	0.0 (0/2)	0.0 (0/2)	0.0 (0/2)	0.0 (0/2)	0.0 (0/2)
Tanzania	*Varanus exanthematicus* (1)	*Amblyomma exornatum*	U	0.0 (0/1)	0.0 (0/1)	0.0 (0/1)	0.0 (0/1)	0.0 (0/1)	0.0 (0/1)
			M	0.0 (0/1)	0.0 (0/1)	0.0 (0/1)	0.0 (0/1)	0.0 (0/1)	0.0 (0/1)
			F	0.0 (0/1)	0.0 (0/1)	0.0 (0/1)	0.0 (0/1)	100.0 (1/1)	0.0 (0/1)
Togo	*Python regius* (U)	*Amblyomma latum*	M	0.0 (0/3)	0.0 (0/3)	0.0 (0/3)	0.0 (0/3)	0.0 (0/3)	0.0 (0/3)
Zambia	*Geochelone paradalis* (9)	*Amblyomma sparsum*	M	70.0 (7/10)	70.0 (7/10)	70.0 (7/10)	0.0 (0/10)	20.0 (2/10)	0.0 (0/10)
			N	37.5 (3/8)	37.5 (3/8)	25.0 (2/8)	25.0 (2/8)	25.0 (2/8)	0.0 (0/8)
**Central and South America**									
Panama	*Bufo marinus* (1)	*Amblyomma rotundatum*	F	100.0 (2/2)	100.0 (2/2)	0.0 (0/2)	0.0 (0/2)	0.0 (0/2)	0.0 (0/2)
Suriname	*Iguana iguana* (3)	*Amblyomma dissimile*	F	0.0 (0/3)	0.0 (0/3)	0.0 (0/3)	0.0 (0/3)	0.0 (0/3)	0.0 (0/3)
**Near and Middle East**									
Jordan	*Geochelone elegans* (U)	*Amblyomma clypeolatum*	M	0.0 (0/2)	0.0 (0/2)	0.0 (0/2)	0.0 (0/2)	0.0 (0/2)	0.0 (0/2)
			N	100.0 (1/1)	100.0 (1/1)	100.0 (1/1)	0.0 (0/1)	0.0 (0/1)	0.0 (0/1)
	*Testudo graeca* (5)	*Hyalomma aegyptium*	M	0.0 (0/1)	0.0 (0/1)	0.0 (0/1)	0.0 (0/1)	0.0 (0/1)	0.0 (0/1)
			F	50.0 (1/2)	50.0 1/2)	50.0 (1/2)	0.0 (0/2)	50.0 (1/2)	0.0 (0/2)
			N	0.0 (0/9)	0.0 (0/9)	0.0 (0/9)	0.0 (0/9)	11.1 (1/9)	0.0 (0/9)
**Central Asia**									
Russia	*Testudo horsfieldii* (1)	*Hyalomma aegyptium*	F	100.0 (2/2)	0.0 (0/2)	0.0 (0/2)	0.0 (0/2)	0.0 (0/2)	0.0 (0/2)
Uzbekistan	*Testudo horsfieldii* (2)	*Hyalomma aegyptium*	M	100.0 (1/1)	100.0 (1/1)	100.0 (1/1)	0.0 (0/1)	0.0 (0/1)	0.0 (0/1)
			F	100.0 (1/1)	100.0 (1/1)	100.0 (1/1)	0.0 (0/1)	0.0 (0/1)	0.0 (0/1)
**South Asia**									
Sri Lanka	*Boiga forsteni* (U)	*Amblyomma trimaculatum*	M	0.0 (0/5)	0.0 (0/5)	0.0 (0/5)	0.0 (0/5)	0.0 (0/5)	0.0 (0/5)
			F	57.1 (8/14)	57.1 (8/14)	57.1 (8/14)	0.0 (0/14)	0.0 (0/14)	0.0 (0/14)
			N	0.0 (0/2)	0.0 (0/2)	0.0 (0/2)	0.0 (0/2)	0.0 (0/2)	0.0 (0/2)
Total				45.2 (42/93)	40.9 (38/93)	23.7 (22/93)	2.2 (2/93)	7.5 (7/93)	0.0 (0/93)

*Number of animal in parenthesis, U: unknown.

**M: male, F: female, N: nymph, U: unknown.

### Polymerase chain reaction (PCR) and sequencing

From the dead ticks, total DNA of the whole body was extracted using the DNeasy tissue kit (Qiagen, Valencia, CA, USA), according to the manufacturer’s instructions. By contrast, the live ticks were dissected, and the salivary gland, mid-gut, and other unidentified tissues, presumably remnants of the excised organs, were individually subjected to DNA extraction.

The PCR primers used in this study are summarized in Table 2. To detect *Rickettsia* species, the genus-common 17-kDa antigen gene (17-kDa antigen), citrate synthase gene (*gltA*), and spotted fever group (SFG)-specific 190-kDa outer membrane protein A gene (*ompA*) were targeted. The 17-kDa antigen primers are specific to the spotted fever and typhus group of *Rickettsia* [10], *gltA* primer sequences were derived from *R*. *prowazekii* and targeted to all species of *Rickettsia* [11], and the *ompA* primer sequences were derived from the reported *R*. *rickettsii* gene and targeted to the SFG *Rickettsia* [12]. For *Ehrlichia* species, the heat shock protein gene (*groEL*) and major outer membrane protein gene (*omp-1*) were used. The *groEL* primers target *Ehrlichia* sp. [13], and the *omp-1* primers are specific to the *omp-1* multigene family of monocytic ehrlichiosis agents [14]. For *Anaplasma*, the gene encoding the outer membrane protein (*p44)* was used, and primers were designed based on the conserved regions of the multigene family *p44* genes of *A*. *phagocytophila* [15]. PCR was performed as described previously [10–15]. PCR amplicons were directly sequenced using the BigDye Terminator v3.1 cycle sequencing kit (Applied Biosystems, Foster City, CA, USA) in an ABI prism 3130 Genetic Analyzer (Applied Biosystems). Sequenced DNA alignments were named in the following order: country of origin, tick species initials, serial number allocated in the study, and genus (-R for *Rickettsia* and -E for *Ehrlichia*). For the dissected tick samples, miscellaneous tissues without the salivary gland and mid-gut were labeled “O,” salivary glands were labeled “S,” and the mid-gut was labeled “M.” Sequenced data were assembled using ATCG software (Genetix, Tokyo, Japan), and the identifications were compared with the GenBank database using the BLAST program (http://blast.ncbi.nlm.nih.gov/Blast.cgi).

**Table 2:** Primers used in PCR and sequencing.

Target		Primer	Primer sequence*	Reference
Tick	mt-*rrs*	mt-rrs(1)	CTGCTCAATGATTTTTTAAATTGCTGTGG	[8]
		mt-rrs(2)	CCGGTCTGAACTCAGATCAAGTA	
	*12S rDNA*	T1B	AAACTAGGATTAGATACCCT	[9]
		T2A	AATGAGAGCGACGGGCGATGT	
*Rickettsia*	17-kDa antigen	R1	TCAATTCACAACTTGCCATT	[10]
		R2	TTTACAAAATTCTAAAAACC	
	*gltA*	RpCs877p	GGGGGCCTGCTCACGGCGG	[11]
		RpCs1258n	ATTGCAAAAAGTACAGTGAAC	
	*ompA*	Rr190.70p	ATGGCGAATATTTCTCCAAAA	[12]
		Rr190.602n	AGTGCAGCATTCGCTCCCCCT	
*Ehrlichia*	*groEL*	gro607F^a^	GAAGATGCWGTWGGWTGTACKGC	[13]
		gro1294R^a^	AGMGCTTCWCCTTCWACRTCYTC	
		gro677F^b^	ATTACTCAGAGTGCTTCTCARTG	
		gro1121R^b^	TGCATACCRTCAGTYTTTTCAAC	
	*omp-1*	conP28-F1^a^	ATYAGTGSAAARTAYRTRCCAA	[14]
		conP28-R1^a^	TTARAARGYAAAYCTKCCTCC	
		conP28-F2^b^	CAATGGRWGGYCCMAGARTAG	
		conP28-R2^b^	TTCCYTGRTARGMAAKTTTAGG	
*Anaplasma*	*p44*	p3726^a^	GCTAAGGAATTAGCTTATGA	[15]
		p4257^a^	AGAAGATCATAACAAGCATTG	
		p3761^b^	CTGCTCKGCCAARACCTC	
		p4183^b^	CAATAGTYTTAGCTAGTAACC	

*: M = A,C; W = A,T; K = G,T; R = A,G; S = C,G; Y = C,T.

^a:^ 1st PCR.

^b:^ nested PCR.

### Accession numbers

The DNA sequences were deposited into the DNA Data Bank of Japan (DDBJ), and accession numbers were obtained as listed in S1 and S2 Tables.

### Phylogenetic analysis

Phylogenetic analysis was performed using CLUSTAL-W with MEGA5.2.2 software (www.megasoftware.net) [16]. The phylogenetic tree was constructed using the neighbor-joining method, and bootstrap tests (1000 replicates) were carried out according to the Kimura 2-parameter method [17]. Pairwise alignments were performed with an open-gap penalty of 10 and a gap extension penalty of 0.5. Multiple alignments were also performed using the same values. All positions containing alignment gaps and missing data were eliminated during the pairwise sequence comparison (pairwise deletion).

## Results

### Ticks and host animals

A total of 93 ticks spanning nine species were collected from at least 28 animals of 10 species. Tick information, host species, and origin countries are summarized in Table 1. All ticks were found attached and feeding on the animals.

### *Rickettsia* and *Ehrlichia* within ticks detected by PCR

The rickettsial genes 17-kDa antigen, *gltA*, and *ompA* were detected in 42, 38, and 22 ticks, respectively (Table 1). Except in ticks originating from Russia, 2 or 3 of the rickettsial genes were co-detected. The *Ehrlichia omp-1* and *groEL* genes were detected in 2 and 7 ticks, respectively (Table 1). The *Anaplasma p44* gene was not detected in any sample. The negative controls for all PCRs were negative.

A snake (*Python regius*) from Ghana had two tick species (*Amblyomma latum* and *Amblyomma transversale*), and only *A*. *transversale* was PCR-positive for *Rickettsia* genes. The tick *A*. *latum* was also carried in the same snake species from Togo, and this tick species was PCR-negative for all genes tested. Snakes (*Boiga forsteni*) from Sri Lanka sampled from a single breeding facility had one tick species (*Amblyomma trimaculatum*), which were PCR-positive for *Rickettsia*. Tortoises (*Geochelone paradalis*) from Africa all carried the same tick species (*Amblyomma sparsum*), but only ticks from Zambia were PCR-positive for both *Rickettsia* and *Ehrlichia*. Tortoises from Jordan, *Geochlelone elegans* and *Testudo graeca*, carried the ticks *Amblyomma clypeolatum* and *Hyalomma aegyptium*, respectively; *Rickettsia* was positively identified in both tick species, but *Ehrlichia* was only positive in *H*. *aegyptium*. Tortoises (*Testudo horsfieldii*) from central Asia carried a single tick species (*H*. *aegyptium*), which was PCR-positive for *Rickettsia*. A gecko (*Phelsuma dubia*) from Madagascar had an *A*. *latum* tick that was PCR-positive for *Rickettsia*, which may be specific to this species or area, since *A*. *latum* in other animals from other countries were all PCR-negative. A lizard (*Varanus exanthematicus*) from Tanzania had *Amblyomma exornatum* ticks, and was positive for *Ehrlichia*. Another lizard species (*Iguana iguana*) had *Amblyomma dissimile* ticks, which were all PCR-negative. A toad (*Bufo marinus*) from Panama carried *Amblyomma rotundatum*, which were PCR-positive for *Rickettsia* genes.

### Sequencing and phylogenetic analysis of rickettsial and ehrlichial genes

All 51 *Rickettsia* 17-kDa antigen gene fragments, 36 of 47 *gltA* gene fragments, 6 of 26 *ompA* gene fragments, and all 7 *Ehrlichia groEL* gene fragments were successfully sequenced (S1 and S2 Tables).

**Fig 1:**
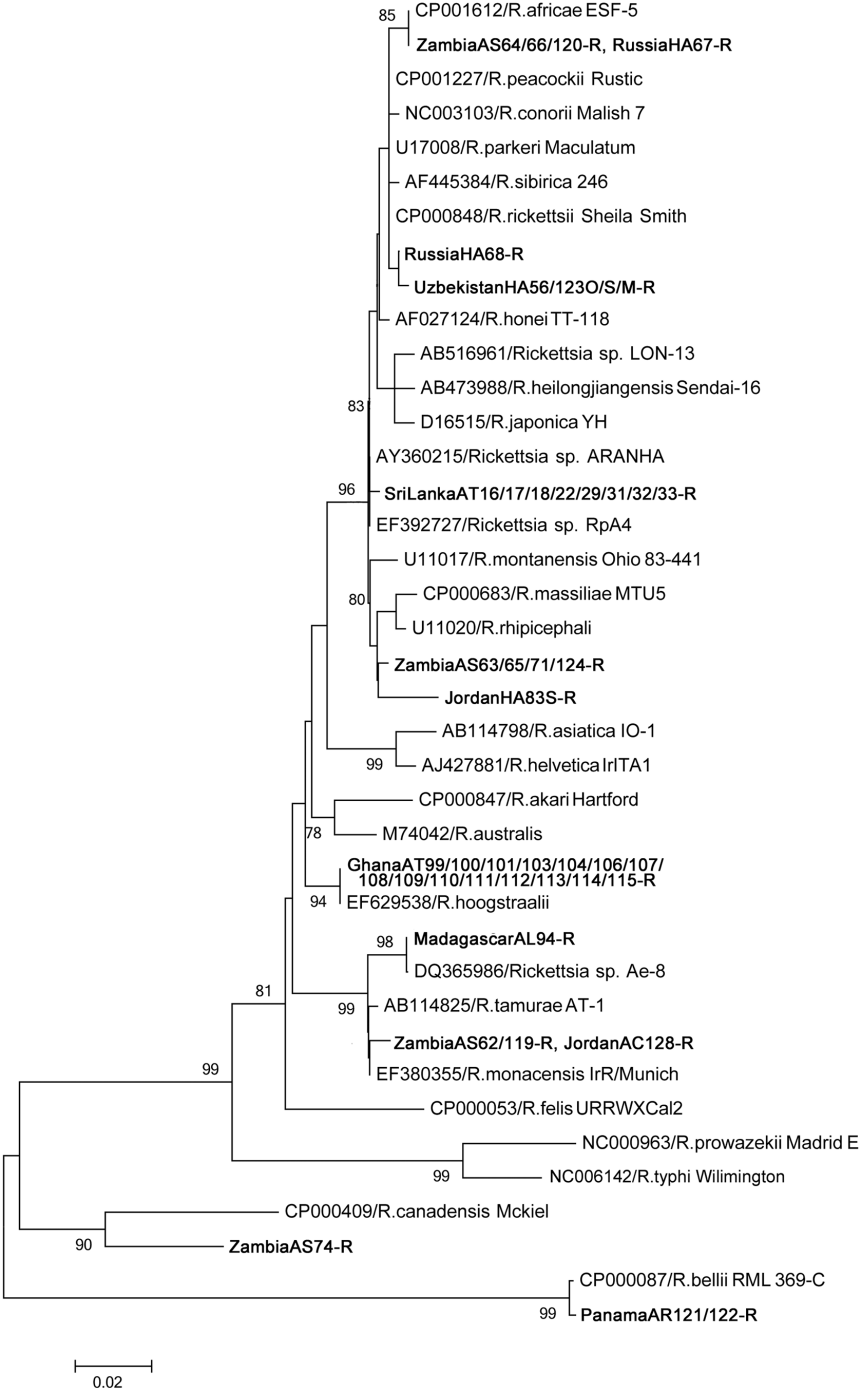
Phylogenetic relationships between the *Rickettsia* spp. genes based on sequence comparison of the 17-kDa antigen gene 394-bp fragment. The phylogenetic branches showed support of >70% by bootstrap analysis. Identified sequences are in bold type. The bar indicates the percentage of sequence divergence.

**Fig 2:**
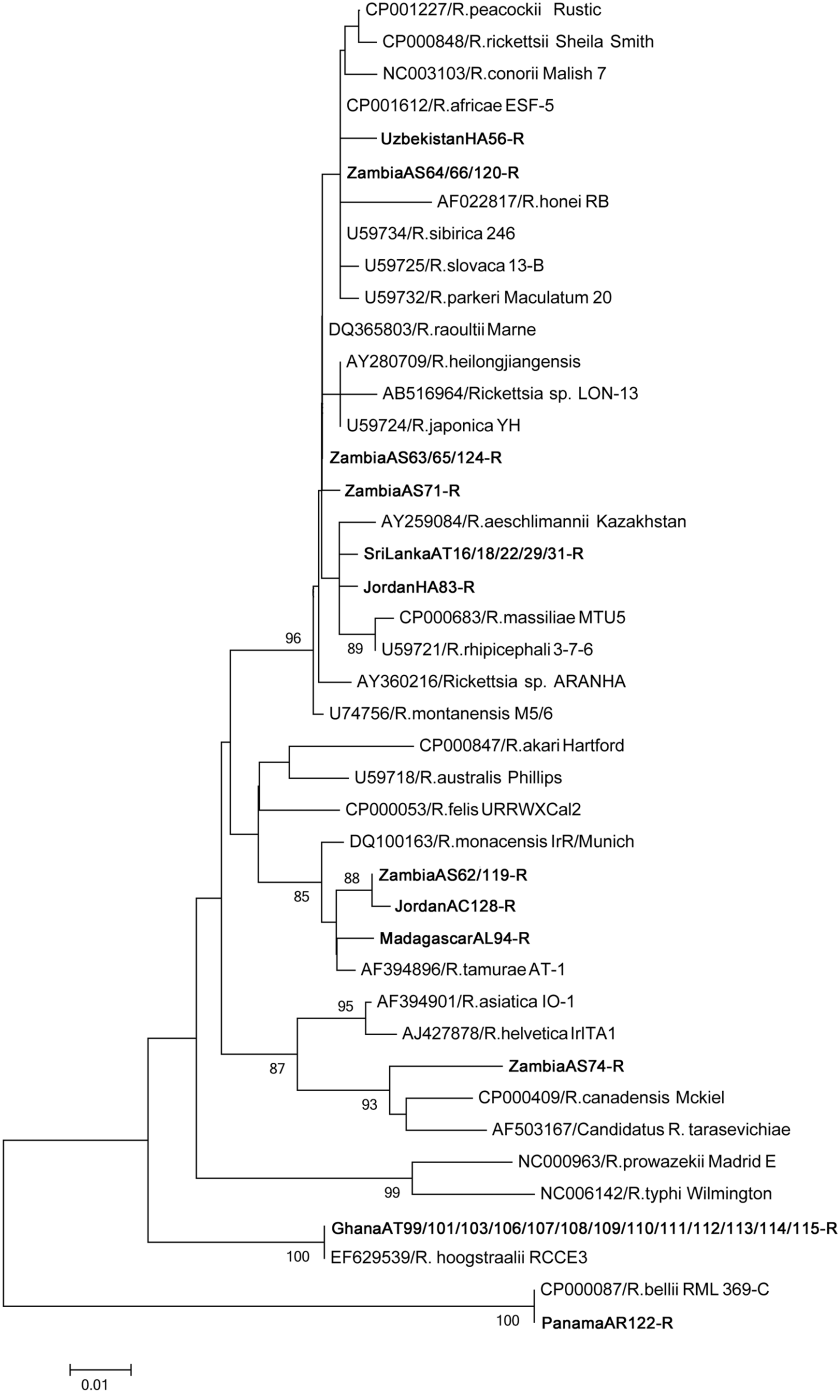
Phylogenetic relationships between the *Rickettsia* spp. genes based on sequence comparison of the *gltA* gene 341-bp fragment. The phylogenetic branches showed support of >70% by bootstrap analysis. Identified sequences are in bold type. The bar indicates the percentage of sequence divergence.

**Fig 3:**
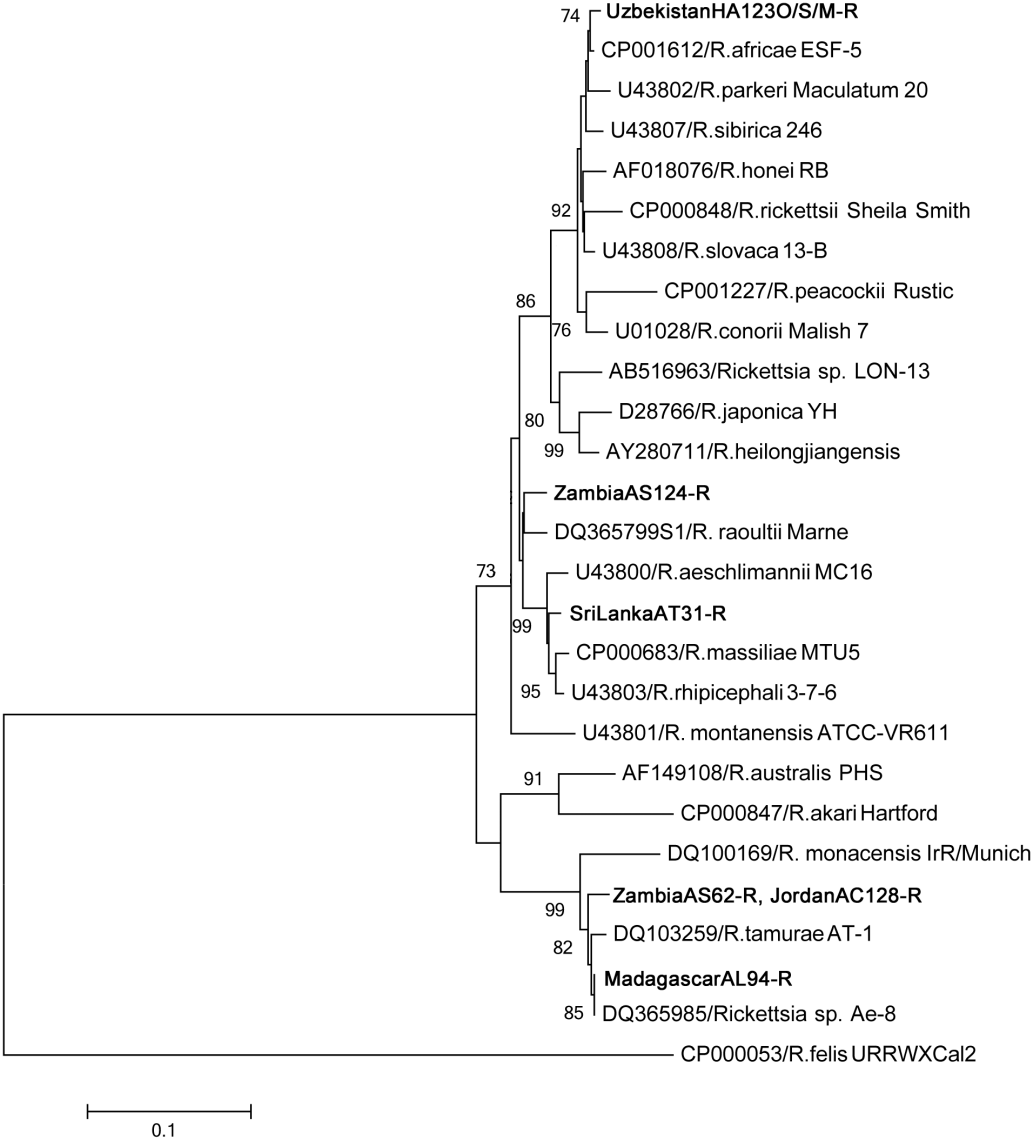
Phylogenetic relationships between the *Rickettsia* spp. genes based on sequence comparison of the *ompA* gene 488-bp fragment. The phylogenetic branches showed supported of >70% by bootstrap analysis. Identified sequences are in bold type. The bar indicates the percentage of sequence divergence.

Phylogenetic analysis of the SFG showed high diversity in the detected rickettsial genes, except for those originating from Panama, which were phylogenetically placed in the ancestral group (Figs 1, 2 and 3). Many of the detected genes showed 100% identity to known *Rickettsia* species; all Ghana isolates and *Rickettsia hoogstraalii* (17-kDa antigen and *gltA*), MadagascarAL94-R and *Rickettsia* sp. Ae-8 (17-kDa antigen and *ompA*), ZambiaAS64/66/120-R and *Rickettsia africae* (17-kDa antigen and *gltA*), ZambiaAS63/65/124-R and *Rickettsia raoultii* (*gltA*), PanamaAR121/122-R and *Rickettsia belli* (17-kDa antigen and *gltA*), and RussiaHA67-R and *R*. *africae* (17-kDa antigen).

Other rickettsial DNA sequences were new and widely located in the SFG *Rickettsia* phylogenetic clusters (Figs 1, 2 and 3). ZambiaAS63/65/71/124-R and JordanHA83-R showed high similarity in 17-kDa (97.9%) and *gltA* (99.1%). ZambiaAS62/119-R and JordanAC128-R are possibly same species, since 17-kDa and *ompA* were 100% identical and *gltA* was 99.7% similar.

**Fig 4:**
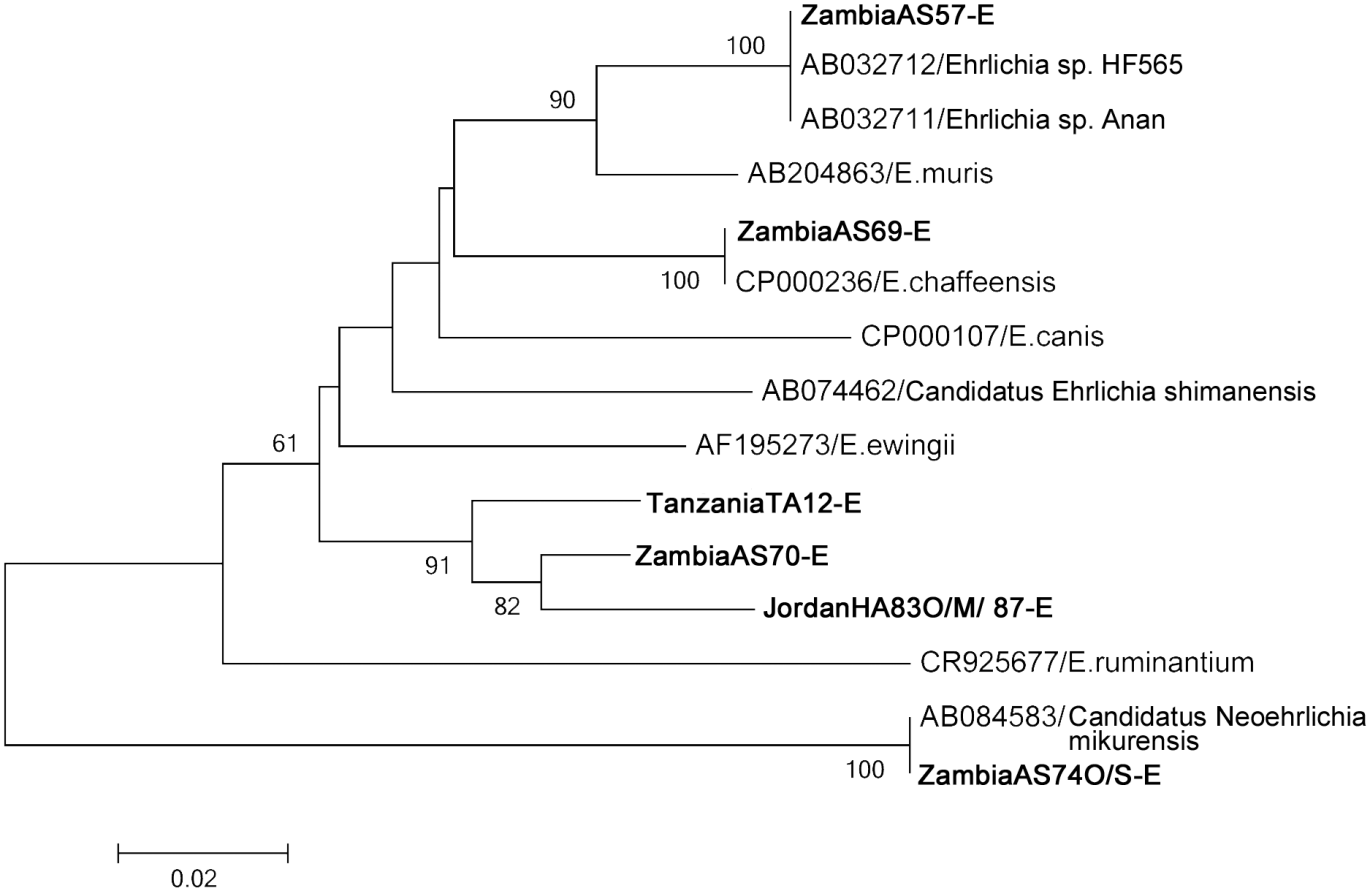
Phylogenetic relationships between the *Ehrlichia* spp. genes based on sequence comparison of the *groEL* gene 319-bp fragment. The phylogenetic branches showed supported of >70% by bootstrap analysis. Identified sequences are in bold type. The bar indicates the percentage of sequence divergence.

In the *Ehrlichia groEL* gene (Fig 4), 100% identity was found between ZambiaAS57-E, *Ehrlichia* sp. HF565, and *Ehrlichia* sp. Anan; ZambiaAS69-E and *Ehrlichia chaffeensis*; and ZambiaAS74O/74S-E and *Candidatus* Neoehrlichia mikurensis. Other new ehrlichial sequences were in the same phylogenetic cluster (Fig 4).

## Discussion

This study revealed that rickettsial and ehrlichial organisms are transported into Japan from abroad through imported exotic animals. The isolates we detected included agents that have known potential to infect humans and animals. The international migration of rickettsial and ehrlichial pathogens is not only a problem for Japan but is also an urgent global issue as the numbers of reported cases are increasing dramatically [1]. There is currently insufficient monitoring of the export and import of reptiles and amphibians both in Japan and elsewhere, and information required to enforce monitoring is lacking.

The rickettsial genes were detected at comparatively high rates in reptile- and amphibian-associated ticks; by contrast, the *Ehrlichia*-specific genes were detected at low rates, and the *Anaplasma*-specific gene was not detected at all in this study. There was no correlation in tick, host, and country of origin for the positivity of multiple genes. The difference in detection rates of each PCR was probably due to differences in the sensitivity of the primer sets. However, our target was not restricted to pathogens with known sequences but rather to a wide variety of Rickettsiaceae and Anaplasmataceae species that may have variable sequences; therefore, it is difficult to evaluate the sensitivity of detection of each PCR. Importantly, many of the detected rickettsial and ehrlichial genes were highly similar to genes of known human and animal pathogens. Thus, the detection of pathogenic organisms in ticks feeding on imported animals that are exempt from quarantine has impacts for both human and veterinary medicine.

Isolates from Ghana all had identical sequences, and showed 100% similarity to *Rickettsia hoogstraalii*, which was detected only from *A*. *transversale* but not from *A*. *latum*. These ticks were collected from a single host animal, suggesting that *A*. *transversale* is a specific tick host for *Rickettsia*. This result shows that *R*. *hoogstraalii* has a wide tick host affinity and geographical distribution, as previously reported for *Haemaphylus sulcata* from Croatia and *Carios capensis* from the United States [18]. Similar sequences to *R*. *hoogstraalii* have been detected in Japan from *C*. *capensis* (accession number AB242434). The pathogenicity of *R*. *hoogstraalii* has not been reported.

Isolates from Zambia, found in one tick species from one host animal species, had a variety of sequences in both the *Rickettsia* and *Ehrlichia* genes. Interestingly, most of the human pathogens detected in this study were from Zambia. We identified four human pathogens in ticks from Zambia: *Rickettsia africae*, *Rickettsia raoultii*, *Ehrlichia chaffeensis*, and *Candidatus* Neoehrlichia mikurensis. Infections of the two *Rickettsia* species were not reported in Japan, except from imported cases, whereas the two *Ehrlichia* species were previously detected in Japan but only in animals [13, 19–21]. *R*. *africae* causes African tick bite fever, a disease characterized by fever and multiple eschars [22]. Although relatively mild and not known to cause mortality, African tick bite fever, which is endemic in sub-Saharan Africa and the French West Indies, is an important emerging disease and is one of the most common forms of rickettsiosis in travel medicine [22]. African tick fever is transmitted principally by the ticks *A*. *variegatum* and *A*. *hebraeum*; in this study, genes from *A*. *sparsum* originating from Zambia showed similarity to the genes of *R*. *africae*, which suggests variation in the vector of transmission. Interestingly, *H*. *aegyptium* of Russia had a 17-kDa antigen fragment similar to that of *R*. *africae*, which may indicate the presence of a unique *Rickettsia* species. *R*. *raoultii* is pathogenic to humans, with cases of infection with eschar-associated spotted fever and tick-borne lymphadenopathy described in previous reports [23, 24]. *E*. *chaffeensis* is the causal agent of human monocytic ehrlichiosis, an acute febrile systematic illness [25]. *Candidatus* Neoehrlichia mikurensis is also known to cause febrile disease in humans [26]. These two *Ehrlichia* species are known to infect mammals [20, 21] but not in humans in Japan.

Genes from other potentially pathogenic organisms also showed high similarity to those isolated from the ticks in this study. *Ehrlichia* sp. HF565 and *Ehrlichia* sp. Anan, which showed 100% identity to an isolate originating from Zambia (ZambiaAS57-E), may potentially be pathogenic to humans based on their close genetic relationship to *E*. *chaffeensis* [27]. An isolate originating from Madagascar was 100% identical to *Rickettsia* sp. Ae-8, a strain of unknown pathogenicity isolated from *A*. *exornatum* in the United States, and is closely related to *R*. *tamurae* [28], which is reportedly pathogenic in humans [29]. The tick *Amblyomma rotundatum* isolated from a toad (*Bufo marinus*) originating from Panama had 100% similar sequences to *Rickettsia bellii*. *R*. *bellii* does not belong to the SFG but rather to an ancestral group, and although its pathogenicity to humans has not been reported, animal infection is known to occur [30]. *R*. *bellii* has been reported from many different tick species in the Americas [1].

As previously mentioned, we detected pathogens that do not naturally exist in Japan. There has been no report of infection of these pathogens in reptiles and amphibians. However, pathogen carriage in ticks associated with reptiles could represent a serious public health threat to the country. Most of the reptiles and amphibians imported to Japan are speculated to be pet animals that are in close contact with humans or other animals. Therefore, even for the microbes of unknown pathogenicity, importing them through their vectors (ticks) and host animals carries a potential and considerable risk of causing emerging disease.

Although there are no reports on the infection of these agents in reptiles and amphibians, there are reports of human-pathogenic *Rickettsia* detected in reptiles, including *R*. *honei*, *Rickettsia monacensis*, and *Rickettsia helvetica* [3, 31]. Our results also suggest the potential presence of reptile-associated *Rickettsia* and *Ehrlichia* species. The ticks originating from Zambia and Jordan (ZambiaAS62-R and JordanAC128-R, and ZambiaAS71-R and JordanHA83-R) exhibited highly similar sequences in the 17-kDa antigen, *gltA*, and *ompA* genes. Other rickettsial and ehrlichial genes detected in the ticks were also closely related to each other. Phylogenetic analysis indicated that these new rickettsial isolates have potential to be pathogenic to mammals since they were placed within the SFG *Rickettsia* phylogenetic clusters. The sequences we detected in this study may help in the future identification of new *Rickettsia* or *Ehrlichia* species. It would be interesting to perform a comprehensive isolation study from these tick species and potentially reveal the presence of novel reptile-specific *Rickettsia* and *Ehrlichia* and their pathogenicity to mammals.

Of the tick species we examined, *A*. *dissimile* and *H*. *aegyptium* reportedly bite humans [32, 33] and potentially cause disease. The tick *A*. *sparsum* of Africa is one of the main vectors for the heartwater agent *Ehrlichia ruminantium*, as are *A*. *variegatum*, and *A*. *marmoreum* [34, 35]. The tick *A*. *dissimile* was shown to be capable of transmitting *E*. *ruminantum* under laboratory conditions [36]. Heartwater is a serious disease in cattle with high fatality. In the United States, *C*. *ruminantium* was detected in *A*. *sparsum* collected from tortoises imported from Africa [37]. In the present study, we detected genes in *A*. *sparsum* that were similar to those in *R*. *africae*, *E*. *chaffeensis*, and *Candidatus* N. mikurensis, which suggests the importance of preventing tick invasion from both a human medical and veterinary perspective.

This study shows the importance of vector surveillance in estimating the risk of emerging zoonoses, especially those causing rickettsial and ehrlichial diseases. However, further studies are needed to verify the prevalence of pathogens in the host animals (reptiles and amphibians) and confirm the risk of disease transmission. For example, evaluation of the tissue or blood samples of hosts and ticks may help to identify vectors and complete the cycle of disease transmission. Based on our results, we recommend establishing a management policy for the imported and exported pathogens or potential pathogens carried by ticks in animals exempt from quarantine in order to improve national and international public health.

## Supporting Information

S1 Table Accession numbers for the detected *Rickettsia spp*. genes. (DOC)

S2 Table Accession numbers for the detected *Ehrlichia spp*. *groEL* gene. (DOC)

## References

[1] Parola P, Paddock CD, Socolovschi C, Labruna MB, Mediannikov O, Kernif T, et al Update on tick-borne rickettsioses around the world: a geographic approach. Clin Microbiol Rev. 2013;26:657–702. 10.1128/CMR.00032-13 24092850 PMC3811236

[2] Randolph SE. The impact of tick ecology on pathogen transmission dynamics In: Bowman AS and Nuttall PA, editors. Ticks. Biology, disease and control. Cambridge: Cambridge University Press; 2008 pp. 40–72.

[3] Stenos J, Graves S, Popov VL, Walker DH. *Aponomma hydrosauri*, the reptile-associated tick reservoir of *Rickettsia honei* on Flinders Island, Australia. Am J Trop Med Hyg. 2003;69:314–317. 14628950

[4] Vaclav R, Ficova M, Prokop P, Betakova T. Associations between coinfection prevalence of *Borrelia lusitaniae*, *Anaplasma* sp., and *Rickettsia* sp. in hard ticks feeding on reptile hosts. Microb Ecol. 2011;61:245–253. 10.1007/s00248-010-9736-0 20711724

[5] Ekner A, Dudek K, Sajkowska Z, Majlathova V, Majlath I, Tryjanowski P. *Anaplasmataceae* and *Borrelia burgdorferi* sensu lato in the sand lizard *Lacerta agilis* and co-infection of these bacteria in hosted *Ixodes ricinus* ticks. Parasit Vectors. 2011;4:182 10.1186/1756-3305-4-182 21933412 PMC3203261

[6] Nieto NC, Foley JE, Bettaso J, Lane RS. Reptile infection with *Anaplasma phagocytophilum*, the causative agent of granulocytic anaplasmosis. J Parasitol. 2009;95:1165–1170. 10.1645/GE-1983.1 19281295

[7] Dantas-Torres F, Oliveira-Filho EF, Soares FA, Souza BO, Valenca RB, Sa FB. Ticks infesting amphibians and reptiles in Pernambuco, Northeastern Brazil. Rev. Bras. Parasitol. Vet. 2008;17:218–221. 19265581 10.1590/s1984-29612008000400009

[8] Ushijima Y, Oliver JH Jr., Keirans JE, Tsurumi M, Kawabata H, Watanabe H, et al Mitochondrial sequence variation in *Carios capensis* (Neumann), a parasite of seabirds, collected on Torishima Island in Japan. J Parasitol. 2003;89:196–198. 12659332 10.1645/0022-3395(2003)089[0196:MSVICC]2.0.CO;2

[9] Takano A, Goka K, Une Y, Shimada Y, Fujita H, Shiino T, et al Isolation and characterization of a novel *Borrelia* group of tick-borne borreliae from imported reptiles and their associated ticks. Environ Microbiol. 2010;12:134–146. 10.1111/j.1462-2920.2009.02054.x 19758349

[10] Anderson BE, Tzianabos T. Comparative sequence analysis of a genus-common rickettsial antigen gene. J Bacteriol. 1989;171:5199–5201. 2768201 10.1128/jb.171.9.5199-5201.1989 PMC210341

[11] Regnery RL, Spruill CL, Plikaytis BD. Genotypic identification of rickettsiae and estimation of intraspecies sequence divergence for portions of two rickettsial genes. J Bacteriol. 1991;173:1576–1589. 1671856 10.1128/jb.173.5.1576-1589.1991 PMC207306

[12] Noda H, Munderloh UG, Kurtti TJ. Endosymbionts of ticks and their relationship to *Wolbachia spp*. and tick-borne pathogens of humans and animals. Appl Environ Microbiol. 1997;63:3926–3932. 9327557 10.1128/aem.63.10.3926-3932.1997 PMC168704

[13] Tabara K, Arai S, Kawabuchi T, Itagaki A, Ishihara C, Satoh H, et al Molecular survey of *Babesia microti*, *Ehrlichia* species and *Candidatus* Neoehrlichia mikurensis in wild rodents from Shimane Prefecture, Japan. Microbiol. Immunol. 2007;51:359–367. 17446675 10.1111/j.1348-0421.2007.tb03923.x

[14] Inayoshi M, Naitou H, Kawamori F, Masuzawa T, Ohashi N. Characterization of *Ehrlichia* species from *Ixodes ovatus* ticks at the foot of Mt. Fuji, Japan. Microbiol Immunol. 2004;48:737–745. 15502406 10.1111/j.1348-0421.2004.tb03599.x

[15] Kim HY, Mott J, Zhi N, Tajima T, Rikihisa Y. Cytokine gene expression by peripheral blood leukocytes in horses experimentally infected with *Anaplasma phagocytophila* . Clin Diagn Lab Immunol. 2002;9:1079–1084. 12204963 10.1128/CDLI.9.5.1079-1084.2002 PMC120081

[16] Tamura K, Dudley J, Nei M, Kumar S. MEGA4: Molecular Evolutionary Genetics Analysis (MEGA) software version 4.0. Mol Biol Evol. 2007;24:1596–1599. 17488738 10.1093/molbev/msm092

[17] Kimura M. A simple method for estimating evolutionary rates of base substitutions through comparative studies of nucleotide sequences. J Mol Evol. 1980;16:111–120. 7463489 10.1007/BF01731581

[18] Duh D, Punda-Polic V, Avsic-Zupanc T, Bouyer D, Walker DH, Popov VL, et al *Rickettsia hoogstraalii* sp. nov., isolated from hard- and soft-bodied ticks. Int J Syst Evol Microbiol. 2010;60:977–984. 10.1099/ijs.0.011049-0 19666817

[19] Kawahara M, Rikihisa Y, Isogai E, Takahashi M, Misumi H, Suto C, et al Ultrastructure and phylogenetic analysis of '*Candidatus* Neoehrlichia mikurensis' in the family Anaplasmataceae, isolated from wild rats and found in *Ixodes ovatus* ticks. Int J Syst Evol Microbiol. 2004;54:1837–1843. 15388752 10.1099/ijs.0.63260-0

[20] Naitou H, Kawaguchi D, Nishimura Y, Inayoshi M, Kawamori F, Masuzawa T, et al Molecular identification of *Ehrlichia* species and '*Candidatus* Neoehrlichia mikurensis' from ticks and wild rodents in Shizuoka and Nagano Prefectures, Japan. Microbiol Immunol. 2006;50:45–51. 16428872 10.1111/j.1348-0421.2006.tb03769.x

[21] Kawahara M, Tajima T, Torii H, Yabutani M, Ishii J, Harasawa M, et al *Ehrlichia chaffeensis* infection of sika deer, Japan. Emerg Infect Dis. 2009;15:1991–1993. 10.3201/eid1512.081667 19961683 PMC3044512

[22] Jensenius M, Fournier PE, Kelly P, Myrvang B, Raoult D. African tick bite fever. Lancet Infect Dis. 2003;3:557–564. 12954562 10.1016/s1473-3099(03)00739-4

[23] Switaj K, Chmielewski T, Borkowski P, Tylewska-Wierzbanowska S, Olszynska-Krowicka M. Spotted fever rickettsiosis caused by *Rickettsia raoultii*—case report. Przegl Epidemiol. 2012;66:347–350. 23101229

[24] Parola P, Rovery C, Rolain JM, Brouqui P, Davoust B, Raoult D. *Rickettsia slovaca* and *R*. *raoultii* in tick-borne Rickettsioses. Emerg Infect Dis. 2009;15:1105–1108. 10.3201/eid1507.081449 19624931 PMC2744242

[25] Rikihisa Y. Clinical and biological aspects of infection caused by *Ehrlichia chaffeensis* . Microbes Infect. 1999;1:367–376. 10602669 10.1016/s1286-4579(99)80053-7

[26] Li H, Jiang JF, Liu W, Zheng YC, Huo QB, Tang K, et al Human infection with *Candidatus* Neoehrlichia mikurensis, China. Emerg Infect Dis. 2012;18:1636–1639. 10.3201/eid1810.120594 23017728 PMC3471638

[27] Shibata S, Kawahara M, Rikihisa Y, Fujita H, Watanabe Y, Suto C, et al New *Ehrlichia* species closely related to *Ehrlichia chaffeensis* isolated from *Ixodes ovatus* ticks in Japan. J Clin Microbiol. 2000;38:1331–1338. 10747103 10.1128/jcm.38.4.1331-1338.2000 PMC86441

[28] Reeves WK, Durden LA, Dasch GA. A spotted fever group *Rickettsia* from an exotic tick species, *Amblyomma exornatum* (Acari: Ixodidae), in a reptile breeding facility in the United States. J Med Entomol. 2006;43:1099–1101. 17017252 10.1603/0022-2585(2006)43[1099:asfgrf]2.0.co;2

[29] Imaoka K, Kaneko S, Tabara K, Kusatake K, Morita E. The first human case of *Rickettsia tamurae* infection in Japan. Case Rep Dermatol. 2011;3:68–73. 10.1159/000326941 21503163 PMC3078220

[30] Pacheco RC, Horta MC, Moraes-Filho J, Ataliba AC, Pinter A, Labruna MB. Rickettsial infection in capybaras (*Hydrochoerus hydrochaeris*) from Sao Paulo, Brazil: serological evidence for infection by *Rickettsia bellii* and *Rickettsia parkeri* . Biomedica. 2007;27:364–371. 18320102

[31] De Sousa R, Lopes de Carvalho I, Santos AS, Bernardes C, Milhano N, Jesus J, et al Role of the lizard *Teira dugesii* as a potential host for Ixodes ricinus tick-borne pathogens. Appl Environ Microbiol. 2012;78:3767–3769. 10.1128/AEM.07945-11 22407681 PMC3346372

[32] Bermudez CS, Castro A, Esser H, Liefting Y, Garcia G, Miranda RJ. Ticks (Ixodida) on humans from central Panama, Panama (2010–2011). Exp Appl Acarol. 2012;58:81–88. 10.1007/s10493-012-9564-7 22544074

[33] Kar S, Dervis E, Akin A, Ergonul O, Gargili A. Preferences of different tick species for human hosts in Turkey. Exp Appl Acarol. 2013;61:349–355. 10.1007/s10493-013-9698-2 23620419

[34] Norval RAI and Mackenzie PKI. The transmission of *Cowdria ruminantium* by *Amblyomma sparsum* . Vet Parasiotol. 1981;8:189–191.

[35] Allsopp BA. Natural history of *Ehrlichia ruminantium* . Vet Parasitol. 2010;167:123–135. 10.1016/j.vetpar.2009.09.014 19836892

[36] Oliver JH Jr, Hayes MP, Keirans JE, Lavender DR. Establishment of the foreign partheonogenetic tick *Amblyomma rotundatum* (Acari: Ixodidae) in Florida. J Parasitol. 1993;79:786–790. 8410557

[37] Burridge MJ, Simmons LA, Simbi BH, Peter TF, Mahan SM. Evidence of *Cowdria ruminantium* infection (heartwater) in *Amblyomma sparsum* ticks found on tortoises imported into Florida. J Parasitol. 2000;86:1135–1136. 11128494 10.1645/0022-3395(2000)086[1135:EOCRIH]2.0.CO;2

