# Supplementary material for: Detection of Rickettsia and Ehrlichia spp. in Ticks Associated with Exotic Reptiles and Amphibians Imported into Japan
Source: PLoS One. 2015 Jul 24;10(7):e0133700. doi: 10.1371/journal.pone.0133700 (PMC4514593; doi:10.1371/journal.pone.0133700)
Supplement: S1 Table — (DOC) [file pone.0133700.s001.doc]

Table S1. Accession numbers for the detected *Rickettsia spp.* genes

| Isolate | 17-kDa antigen gene | *gltA* gene | *ompA* gene |
| --- | --- | --- | --- |
| SriLankaAT16-R | AB795111 | AB795166 |  |
| SriLankaAT17-R | AB795112 |  |  |
| SriLankaAT18-R | AB795113 | AB795167 |  |
| SriLankaAT22-R | AB795114 | AB795168 |  |
| SriLankaAT29-R | AB795115 | AB795169 |  |
| SriLankaAT31-R | AB795116 | AB795170 | AB795202 |
| SriLankaAT32-R | AB795117 |  |  |
| SriLankaAT33-R | AB795118 |  |  |
| UzbekistanHA56-R | AB795128 | AB795174 |  |
| ZambiaAS62-R | AB795132 | AB795175 | AB795205 |
| ZambiaAS63-R | AB795133 | AB795176 |  |
| ZambiaAS64-R | AB795134 | AB795177 |  |
| ZambiaAS65-R | AB795135 | AB795178 |  |
| ZambiaAS66-R | AB795136 | AB795179 |  |
| RussiaHA67-R | AB795137 |  |  |
| RussiaHA68-R | AB795138 |  |  |
| ZambiaAS71-R | AB795139 | AB795180 |  |
| ZambiaAS74-R | AB795140 | AB795181 |  |
| JordanHA83S-R | AB795141 ^b^ | AB795182 ^b^ |  |
| MadagascarAL94-R | AB795142 | AB795183 | AB795206 |
| GhanaAT99-R | AB795143 | AB795184 |  |
| GhanaAT100-R | AB795144 |  |  |
| GhanaAT101-R | AB795145 | AB795185 |  |
| GhanaAT103-R | AB795146 | AB795186 |  |
| GhanaAT104-R | AB795147 |  |  |
| GhanaAT106-R | AB795148 | AB795187 |  |
| GhanaAT107-R | AB795149 | AB795188 |  |
| GhanaAT108-R | AB795150 | AB795189 |  |
| GhanaAT109O/S/M-R | AB795152 b/AB795151 ^c^ | AB795190 ^a^ |  |
| GhanaAT110-R | AB795153 | AB795191 |  |
| GhanaAT111-R | AB795154 | AB795192 |  |
| GhanaAT112-R | AB795155 | AB795193 |  |
| GhanaAT113-R | AB795156 | AB795194 |  |
| GhanaAT114-R | AB795157 | AB795195 |  |
| GhanaAT115-R | AB795158 | AB795196 |  |
| ZambiaAS119-R | AB795159 | AB795197 |  |
| ZambiaAS120-R | AB795160 | AB795198 |  |
| PanamaAR121-R | AB795161 |  |  |
| PanamaAR122-R | AB795162 | AB795199 |  |
| UzbekistanHA123O/S/M-R | AB795163 ^a^ |  | AB795208 b/AB795207 ^c^ |
| ZambiaAS124-R | AB795164 | AB795200 | AB795209 |
| JordanAC128-R | AB795165 | AB795201 | AB795210 |

DNA was isolated from the salivary gland and midgut removed from the whole tick^a^, and the salivary gland^b^ and midgut^c^ alone.
