# Supplementary material for: Detection of Rickettsia and Ehrlichia spp. in Ticks Associated with Exotic Reptiles and Amphibians Imported into Japan
Source: PLoS One. 2015 Jul 24;10(7):e0133700. doi: 10.1371/journal.pone.0133700 (PMC4514593; doi:10.1371/journal.pone.0133700)
Supplement: S2 Table — (DOC) [file pone.0133700.s002.doc]

Table S2. Accession numbers for the detected *Ehrlichia* *spp. groEL* gene*.*

| Isolate | Accession number |
| --- | --- |
| TanzaniaTA12-E | AB795211 |
| ZambiaAS57-E | AB795212 |
| ZambiaAS69-E | AB795213 |
| ZambiaAS70-E | AB795214 |
| ZambiaAS74O/S-E | AB795216 a/AB795215 ^b^ |
| JordanHA83O/M-E | AB795219 a/AB795218 ^c^ |
| JordanHA87-E | AB795220 |

DNA was isolated from salivary gland and mid gut removed from the whole tick^a^, and the salivary gland^b^, and midgut^c^ alone.
